# Development of a 3D-printed nuchal translucency model: a pilot study for prenatal ultrasound training

**DOI:** 10.1007/s00404-024-07561-8

**Published:** 2024-05-25

**Authors:** Florian Recker, Laura Remmersmann, Elena Jost, Jorge Jimenez-Cruz, Nicolas Haverkamp, Ulrich Gembruch, Brigitte Strizek, Valentin S. Schäfer

**Affiliations:** 1 Department of Obstetrics and Prenatal Medicine, University Hospital Bonn, Venusberg Campus 1, 53127 Bonn, Germany; 2 Office of Academic Affairs, University Hospital Bonn, Venusberg Campus 1, 53127 Bonn, Germany; 3 Department of Rheumatology and Clinical Immunology, Clinic of Internal Medicine III, University Hospital Bonn, Bonn, Germany

**Keywords:** First trimester screening, Medical education, Nuchal translucency, 3D printing

## Abstract

**Background:**

We used two 3D ultrasound volumes of fetal heads at 13 weeks to create live-size 3D-printed phantoms with a view to training or assessment of diagnostic abilities for normal and abnormal nuchal translucency measurements. The phantoms are suitable for use in a water bath, imitating a real-life exam. They were then used to study measurement accuracy and reproducibility in examiners of different skill levels.

**Methods:**

Ultrasound scans of a 13 + 0-week fetus were processed using 3D Slicer software, producing a stereolithography file for 3D printing. The model, crafted in Autodesk Fusion360™, adhered to FMF guidelines for NT dimensions (NT 2.3 mm). Additionally, a model with pathologic NT was designed (NT 4.2 mm). Printing was performed via Formlabs Form 3® printer using High Temp Resin V2. The externally identical looking 3D models were embedded in water-filled condoms for ultrasound examination. Eight specialists of varying expertise levels conducted five NT measurements for each model, classifying them in physiological and abnormal models.

**Results:**

Classification of the models in physiological or abnormal NT resulted in a detection rate of 100%. Average measurements for the normal NT model and the increased NT model were 2.27 mm (SD ± 0.38) and 4.165 mm (SD ± 0.51), respectively. The interrater reliability was calculated via the intraclass correlation coefficient (ICC) which yielded a result of 0.883, indicating robust agreement between the raters. Cost-effectiveness analysis demonstrated the economical nature of the 3D printing process.

**Discussion:**

This study underscores the potential of 3D printed fetal models for enhancing ultrasound training through high inter-rater reliability, consistency across different expert levels, and cost-effectiveness. Limitations, including population variability and direct translation to clinical outcomes, warrant further exploration. The study contributes to ongoing discussions on integrating innovative technologies into medical education, offering a practical and economical method to acquire, refine and revise diagnostic skills in prenatal ultrasound. Future research should explore broader applications and long-term economic implications, paving the way for transformative advancements in medical training and practice.

## What does this study adds to the clinical work

**Table:** 

This study significantly enhances clinical practice by introducing a 3D-printed ultrasound NT model for improved training in prenatal screening. The model offers a realistic, cost-effective tool for clinicians to refine NT measurement skills, crucial for detecting chromosomal abnormalities. Its use promises to heighten diagnostic accuracy, essential for effective patient care. Additionally, this innovative approach provides a standardized and accessible training method. The model's accuracy and cost-effectiveness represent a substantial advancement in medical education, potentially transforming training and diagnostic proficiency in prenatal ultrasound.	

What does this study add to the clinical work?

## Introduction

The scientific relevance of nuchal translucency (NT) gained prominence in the 1990s when increased measures for sonographically observable NT were correlated with chromosomal anomalies, notably trisomy 21 [1]. However, subsequent research illuminated the broader spectrum of conditions associated with elevated fetal NT, extending beyond chromosomal disorders to encompass malformations such as those affecting the heart, lungs, skeletal structures, and congenital infections [2]. This realization prompted a shift in clinical practice, introducing more comprehensive ultrasound examinations and invasive procedures when confronted with increased NT during pregnancy.

The multifaceted etiology of elevated NT in both chromosomal and non-chromosomal disorders led to the exploration of various pathomechanisms. Chromosomal abnormalities are hypothesized to modify the composition of extracellular material, and venous congestion is thought to lead to a minor elevation in the pressure of venous and lymphatic outflow [3]. Additionally, conditions such as anemia and malformations of the lymphatic system were found to contribute to increased NT [4].

Accurate interpretation of NT requires adherence to specific gestational age windows (11 + 0–13 + 6 weeks) and crown-rump length (CRL) ranges (45 mm–84 mm) [8]. The NT measurement is intricately linked to CRL values, with variations ranging from 1.2 mm (CRL: 45 mm) to 2.7 mm (CRL: 84 mm) [3]. Physicians commonly identify increased NT [5] when it surpasses the 95th percentile relative to CRL [6].

For valid NT predictions, the Fetal Medical Foundation (FMF) has established criteria for NT measurement, emphasizing factors such as gestational age, CRL range, imaging parameters, and anatomical details. Recognizing the importance of both technique and training in ensuring the validity of NT measurements, the FMF has demonstrated that, with adequate training, differences in NT measurements between different examinations decrease over time [7, 8].

In recent years, the medical field has witnessed the burgeoning application of three-dimensional (3D) printing technology, not only for the visualization of malformations but also as a powerful tool in medical training [9–11] It is therefore not surprising that this technology has also created new innovative areas of application for 3D printing in the field of gynecology and obstetrics [10]. In addition to the educational approach (e.g. models for students, relatives, education [12]) and individualized medical devices (e.g. pessaries [13], dilators [14]), artificial tissues similar to tissue were printed with biological ink and cells, which were used to reconstruct or cover defects in the vagina [15, 16]. Other application examples include the preoperative planning of a caesarean section for uterine myomatosis using a 3D model from MRI data [17]. Preoperative planning for the separation of conjoined twins [18]. The development of a pelvic floor model to improve examination techniques [19].

3D printed models emerge as a cost-effective and efficient alternative for enhancing ultrasound skills [10, 20]. This study aims to design a low-cost ultrasound NT model derived from a healthy fetus, adhering to FMF criteria, to facilitate the training of NT measurement.

## Materials and methods

The following four steps were performed to create a 3D ultrasound model:

### Step 1: creation of a 3D model

**Fig. 1:**
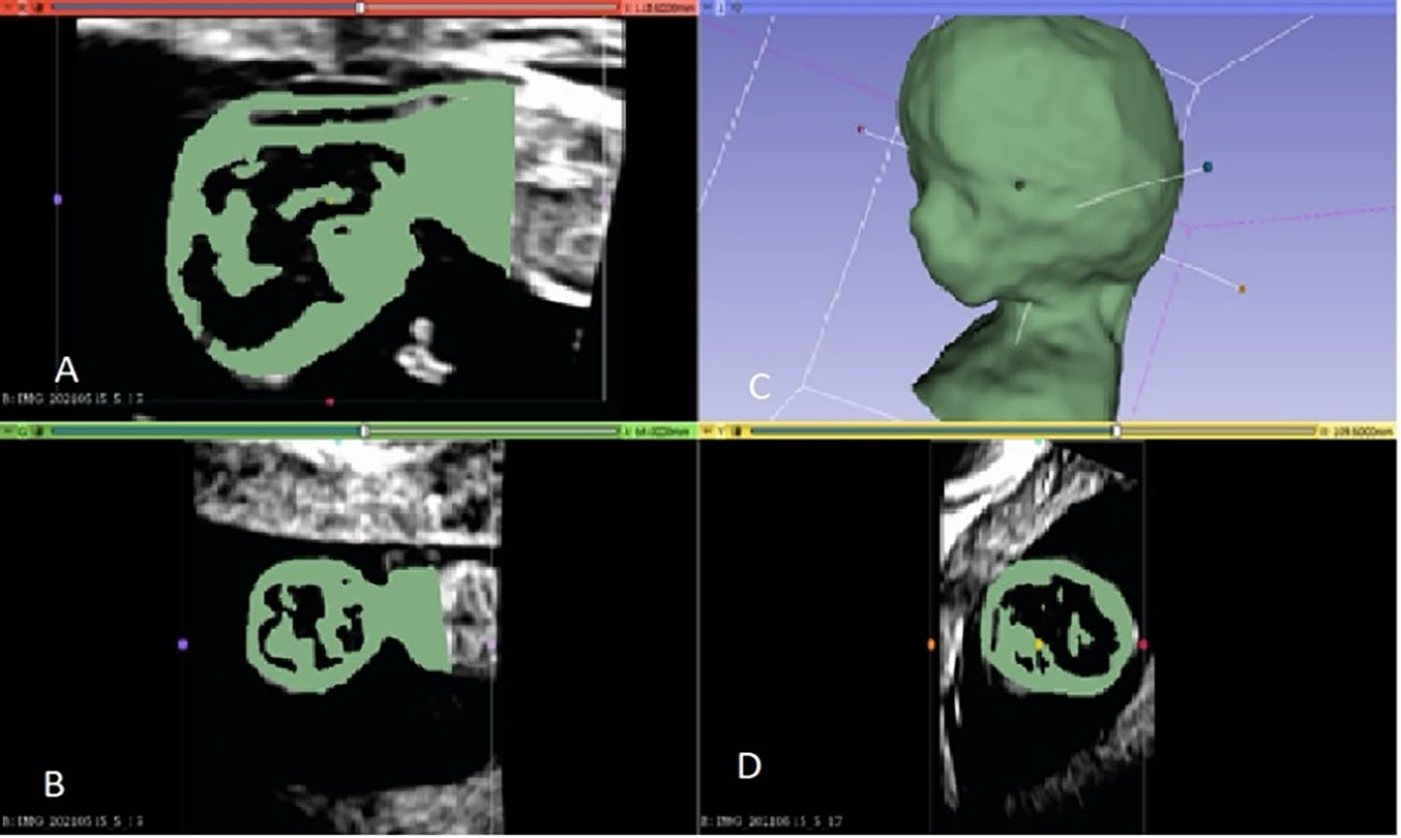
Demonstrating the reconstruction of a 3D model using ultrasound images in three different planes: **A** sagittal, **B** coronal, and **D** transverse. **C** showcases the resulting 3D model computed by the software

Ultrasound scans of a healthy fetus at 13 + 0 weeks of gestation (CRL of 66 mm) were obtained from a healthy pregnant woman under the guidance of an experienced physician trained in the standards set by the FMF, utilizing the Voluson E 10 ultrasound machine (GE Healthcare, Solingen, Germany). The acquired ultrasound images, in Cartesian volume format, were subsequently processed using 3D Slicer software (version 4.11.20210226 r29738/7a593c8). The software facilitated the conversion of these images into a 3D representation of the fetal body. The fetus was then isolated and refined from the uterine background through the application of various image processing tools. The determination of NT involved predefining thresholds, followed by meticulous refinement through the manual editing of each layer. The resultant 3D model, stored as a slicer file, was further transformed into a stereolithography file (.stl) to enable additional enhancements using other software programs. This multi-step process aimed to ensure the accuracy and quality of the 3D representation for further analysis and application in subsequent stages of the research (Fig. 1).

### Step 2: Model improvement for printing

**Fig. 2:**
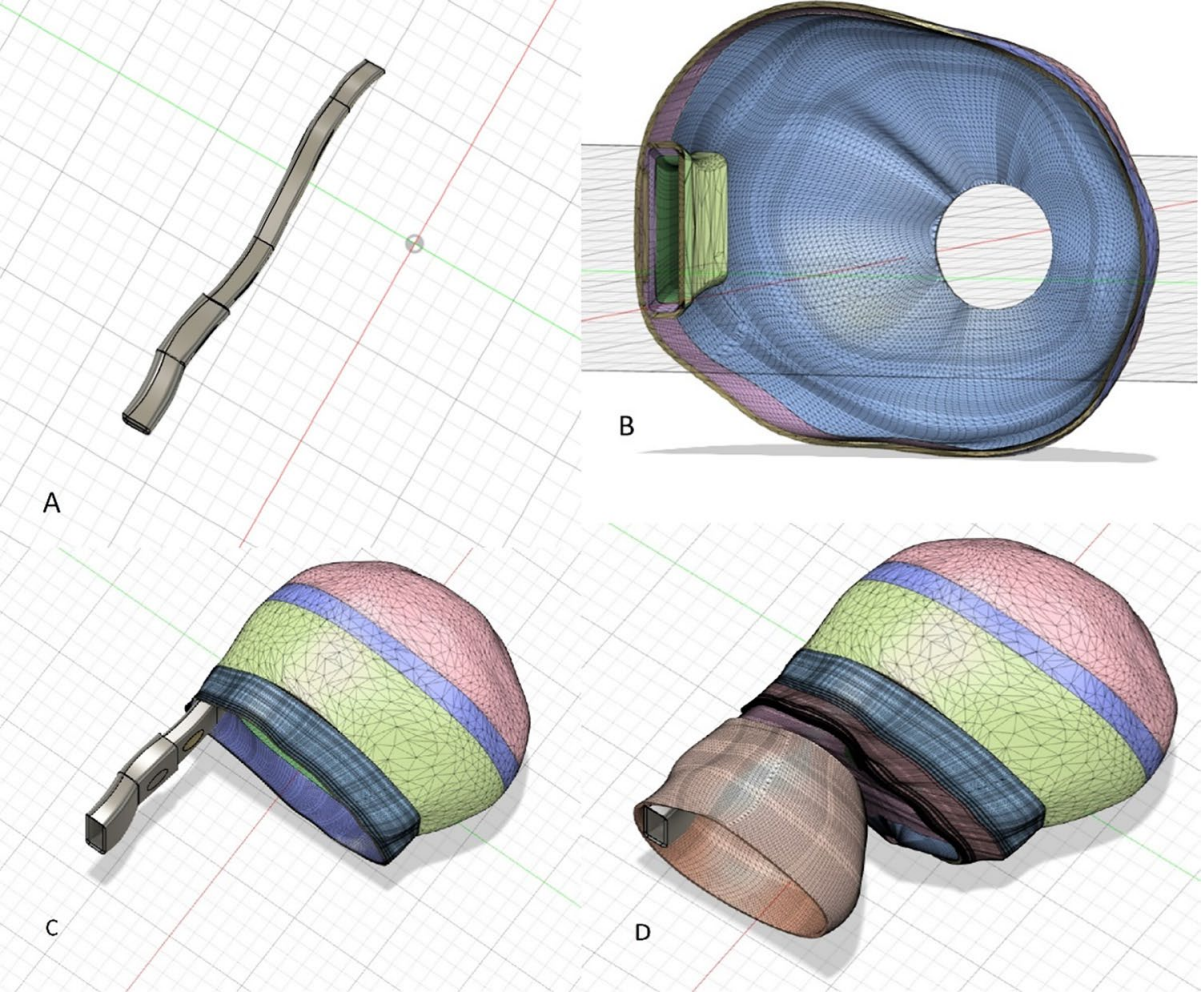
Editing the .stl file in Autodesk’s Fusion 360™ software into parts. **A** reconstructed NT, **B** half skull with the corresponding NT, **C** skull and NT, **D** full model

The obtained .stl file underwent integration into Autodesk's Fusion360™ software (Autodesk Inc., San Rafael, CA, USA), where diverse approaches were explored to achieve the most slender model possible. To achieve this, the pre-processed model generated in 3D Slicer was systematically reconstructed layer by layer, leveraging the foundational model. A notable advantage of this methodology lies in its efficiency—once each layer is defined, subsequent modifications can be executed with a single mouse click (Fig. 2).

In the reconstructed model, the NT segment was shaped into a rectangle with a thickness of 2.3 mm and a wall thickness of 1 mm, resulting in an overall thickness from outer wall to outer wall of 2.5 mm. A second model was crafted with an NT segment of 4.2 mm (plus an additional 1 mm wall thickness in each direction). A wall thickness of 1 mm appeared to yield optimal results in terms of model stability and ultrasound transmission. Subsequently, the model was divided into two segments and specific perforations were incorporated for subsequent procedural steps.

### Step 3: Printing process

**Fig. 3:**
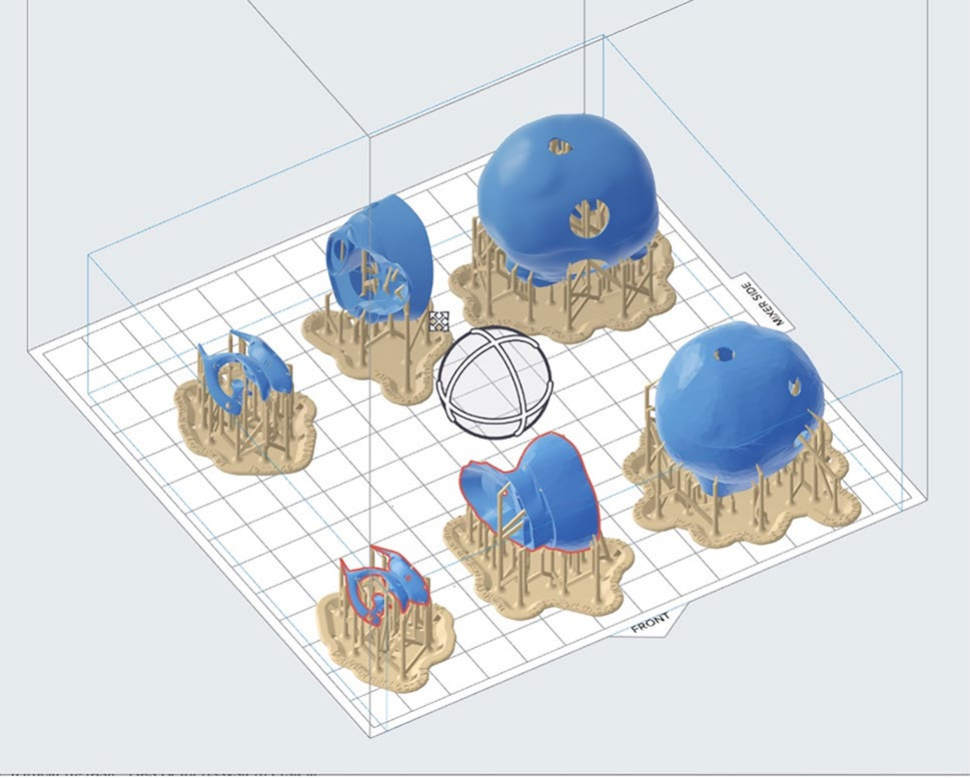
Optimized printing setup in the Formlabs PreForm software showing the individual parts of the model arranged on the printing platform, with the program concurrently calculating the printability of the model

The.stl file underwent integration into the printing software PreForm (Version 3.28.0, Formlabs, Somerville, Massachusetts, USA). To enhance printability, support structures were incorporated into the model (Fig. 3). During the Autodesk processing, the insertion of holes in both the skull and the NT segment became necessary due to error messages related to vacuum. These holes not only address cleaning requirements for the model but also ensure pressure equalization. Subsequent adjustments to the model’s size remained feasible with PreForm.

Printing was executed using the Formlabs Form3 + 3D printer (Formlabs, Somerville, Massachusetts, USA), utilizing specific heat-resistant resin (High Temp Resin V2, Formlabs, Somerville, Massachusetts, USA) with an impressive resolution of 0.025 mm per line. Previously, various types of resin (white resin, flexible resin, and heat-resistant resin) were explored. Among these, the heat-resistant resin emerged as the optimal choice, producing the most satisfactory results. The printer employs low-force stereolithography, a technique triggering a chemical reaction in the resin rather than a layer-by-layer printing process. Essentially, the model is extracted from the bottom of the 3D printer tank, where the chemical reaction occurs. This method minimizes impedances in ultrasound examinations.

**Fig. 4:**
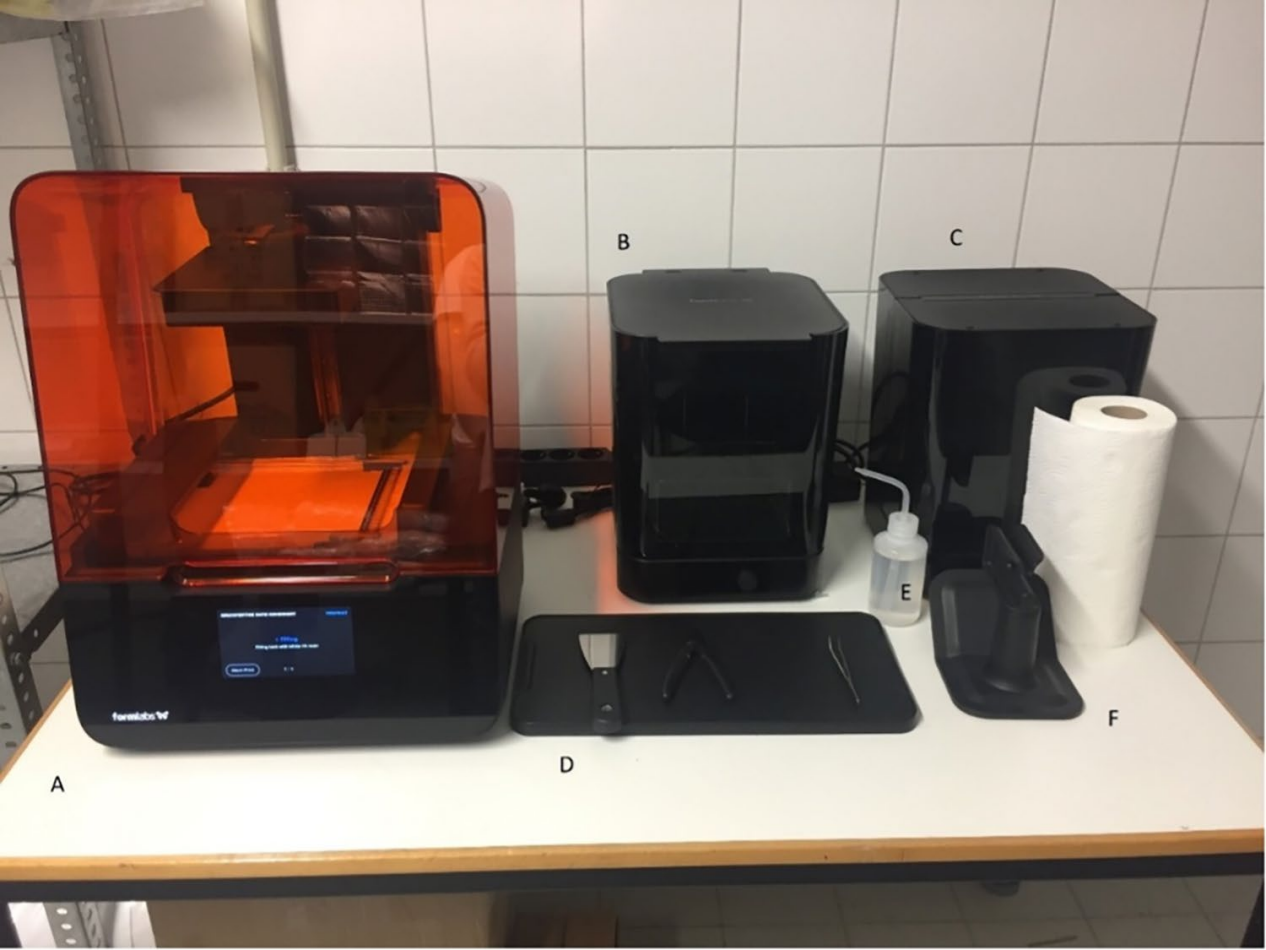
Workplace setup: **A** 3D printer, **B** washing device, **C** curing device, **D** essential tools, **E** isopropyl alcohol for precision cleaning, **F** processing handle

**Fig. 5:**
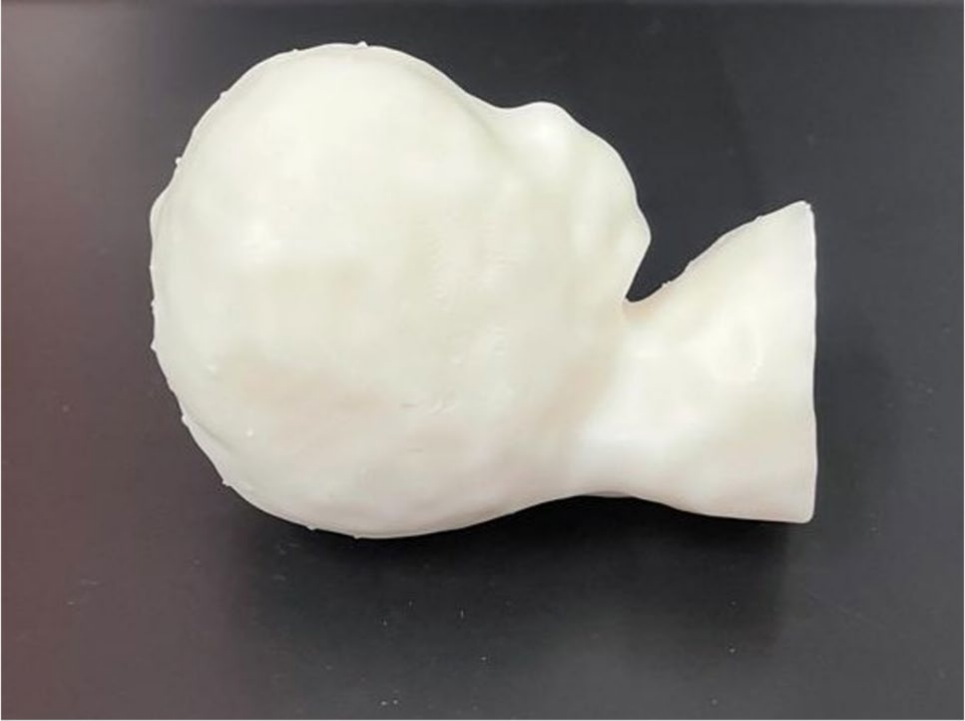
The printed first-trimester fetus

Post-printing, the model required washing with isopropanol alcohol at the Formlabs washing station for 10 min and subsequent curing for 120 min at 60° to achieve the desired final state (Form Wash/Form Cure, Formlabs, Somerville, Massachusetts, USA) (Figs. 4, 5).

### Step 4: Preparation for ultrasound application

**Fig. 6:**
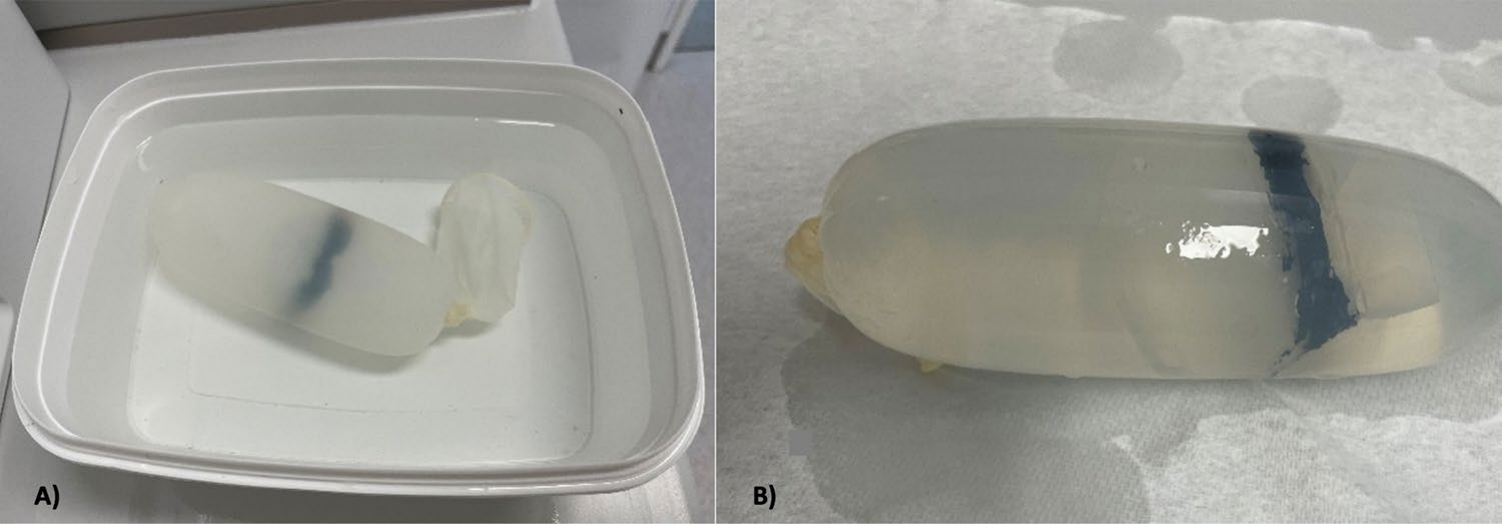
Compact Model Encapsulation: **A** 3D printed model embedded in water-filled condoms immersed in the water tank, **B** Condom containing NT model and water for enhanced visualization

To enable sonographic visibility of the constructed cavities in our model, various embedding solutions were tested including ballistic gelatine, water, and a solution thickened with psyllium husks. Optimal results were achieved through the following procedure: Initially, the two segments of the model were joined using commercial glue. After curing process of 24 h, the assembled models were enveloped in flexible latex protection covers/condoms (European article number 4008600174912, diameter of 34 mm, MAPA GmbH) commonly utilized for vaginal ultrasound probes. The condoms containing the model were then filled with water to eliminate air bubbles. The model was not fixed in the aqueous solution. The previous mentioned intentional introduction of artificial holes in the NT segment and skull facilitated the water-filling process. To further prevent trapping of air bubbles, the water-filled condoms were closed in containers of water (Fig. 6). This method ensured the least occurrence of air bubbles, minimizing artifacts via reduction of impedances in the subsequent ultrasound examination.

### Step 5: Ultrasound examination of the 3D-model

**Fig. 7:**
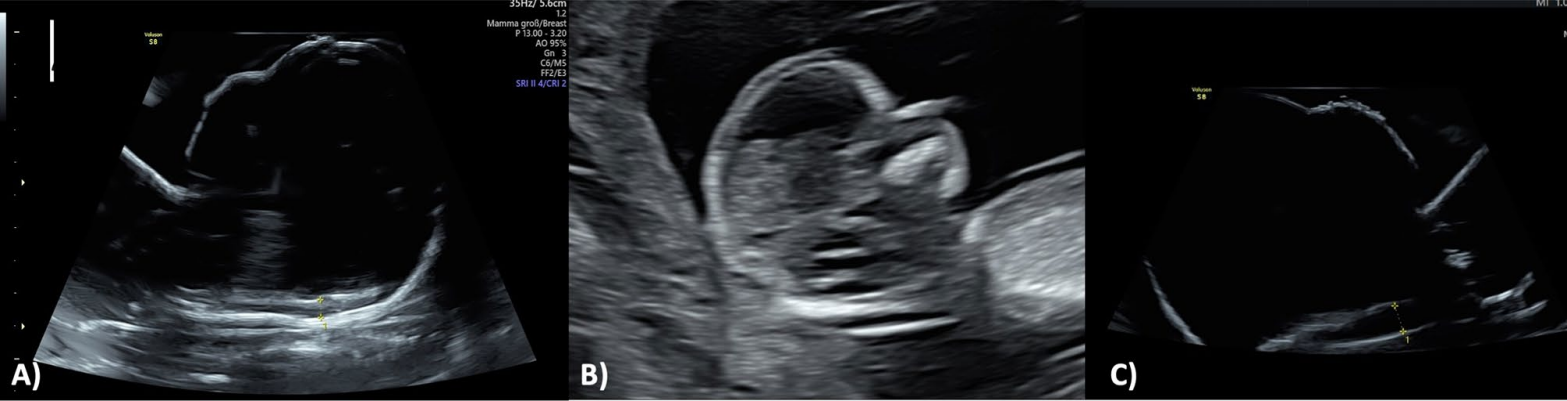
Comparative visualization of the physiological nuchal translucency (NT) in the fetal 3D model (**A**) and in the sonogram of the healthy fetus (13 + 0 weeks of gestation) (**B**) as well as the unphysiological fetal 3D model (**C**)

The ultrasound examination of the model was performed using the Voluson S8 Touch (GE Healthcare, Solingen, Germany) equipped with a linear and curved transducer. This configuration facilitated the acquisition of high-resolution images by capitalizing on the spatial proximity between the transducer and the model. Employing the B-mode with 1–5 MHz and an image penetration depth of 8 cm, measurements were conducted in sagittal sections in millimeters (Fig. 7). A total of eight highly trained ultrasound specialists in prenatal medicine (DEGUM/EFSUMB Level I-III and FMF London) independently conducted five measurements each on both models. External distinctions between the abnormal and physiological models were not discernible. The examiner holds the model with one hand for the measurement and the correct plane is set with the other. As soon as the examiner had set the correct plane in his opinion, the image was frozen on instruction. The examiner then measured the NT independently. Following the completion of the five measurements for each model, the investigators disclosed their assessments regarding which model was physiological or abnormal. The sequence in which the physiological or abnormal model was presented to the examiners for ultrasound assessment was randomly determined by the investigator.

### Model evaluation and statistical analysis

To evaluate the model design, the following measures were examined:

1. Detection rate: How many of the examiners are able to correctly assess the difference between physiological and abnormal models?
2. Inter-rater reliability: To which extent do the examiners agree in their observations?

Statistical analyses and Bland–Altman plots were both performed or created using Microsoft Excel 2016 for Windows 11. For quantitative parameters, statistical measures such as mean, standard deviation (SD), and range were determined. Significant changes were assessed through t-tests, and Spearman correlation analysis was employed. To assess interrater reliability, the intraclass correlation coefficient (ICC) [21] (version 2.1) between the readers was calculated from a two-factor analysis of variance (ANOVA). The interpretation of ICC values followed Rosner’s criteria [17]: ICC < 0.4 denoted poor reliability, 0.4 ≤ ICC < 0.75 indicated fair to good reliability, and ICC ≥ 0.75 suggested excellent reliability [22].

## Results

Our 3D printed fetal models faithfully adhered to the NT criteria outlined by FMF, accurately representing both normal and increased instances of fetal NT in prenatal assessments (Fig. 7).

Our groundbreaking method precisely replicated NT thickness in these models, allowing for accurate measurements of NT values in both standard and atypical conditions.

### Classification of models and inter-rater scoring

All eight examiners correctly classified the models as either physiological or abnormal, resulting in a perfect detection rate of 100% for identification of increased NT. The measured average value for the physiological model, based on assessments from all investigators, was 2.27 mm (SD ± 0.41) in relation to the constructed NT of 2.3 mm. The values for the minimal and maximal assessments were 1.84 mm and 2.96 mm, respectively. In contrast, the measurement of the abnormal model yielded an average value of 4.12 mm (SD ± 0.55) referring to a constructed NT of 4.2 mm. The minimal measurement for this model was 3.1 mm, while the maximal value was 4.9 mm.

**Fig. 8:**
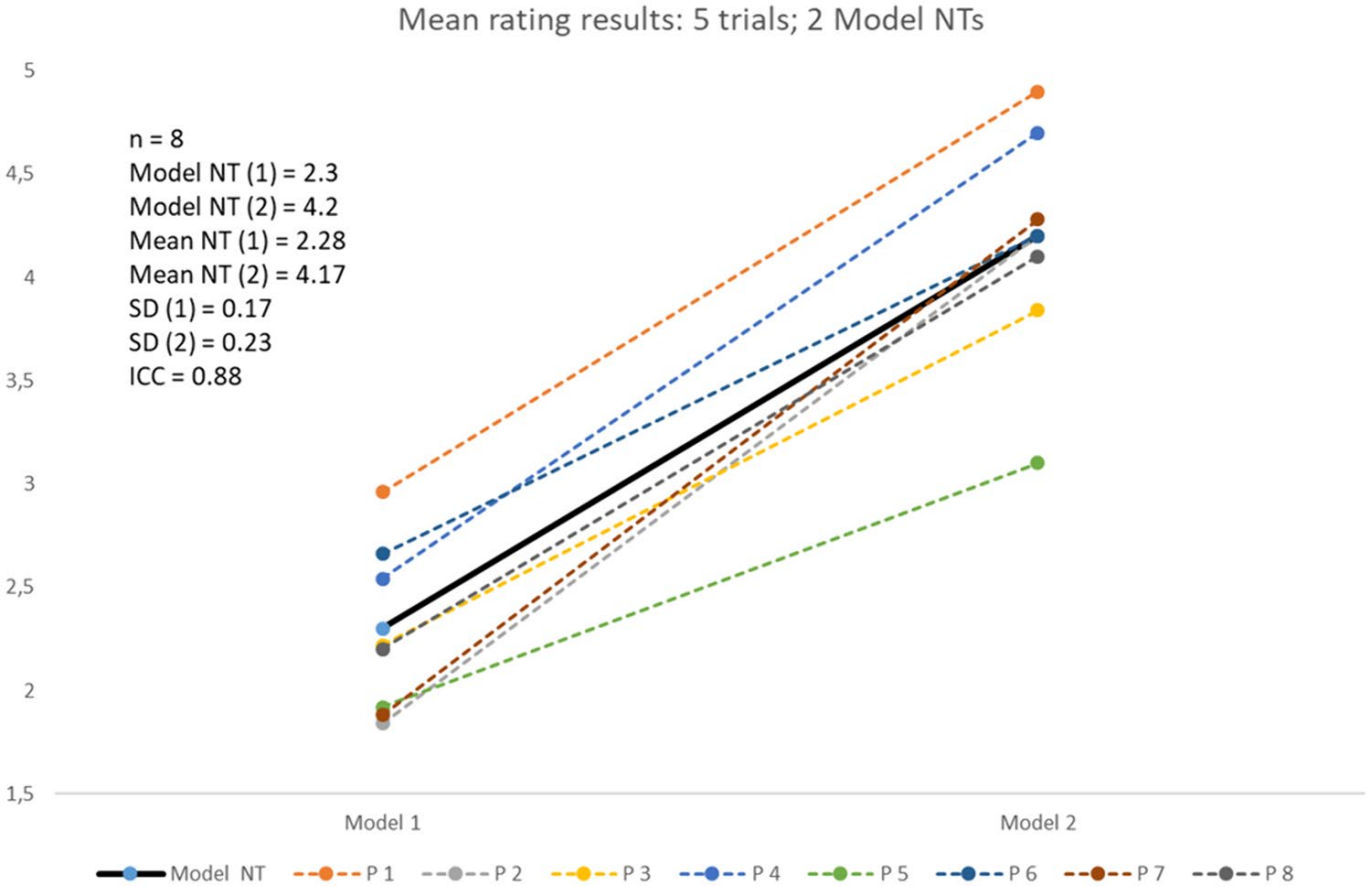
Mean values conducted by the 8 examiners in 5 trials for the 2 models and the resulting intraclass correlation coefficient (ICC)

The mean values per examiner, resulting from five examinations per model, for the two models were consolidated into a single graph and are presented in Fig. 8. This ICC value demonstrates a strong agreement between the eight examiners, with a high coefficient of 0.88 for the mean measurement values. This coefficient denotes an excellent interrater reliability, suggesting consistent judgment across the diverse raters.

### The 3D model and the measurement variance of the investigators

Furthermore, this investigation entailed the meticulous extraction of nuchal translucency measurements from the GE ViewPoint Version 6.0 system (GE Healthcare, Solingen, Germany), which constitutes a pivotal element of the prenatal diagnostic regimen administered by the designated medical practitioners. This extraction was pivotal in delineating the diagnostic precision and variability inherent in routine prenatal screening methodologies.

Subsequent to the data acquisition, a comprehensive analytical process was undertaken. This process involved a systematic comparison and correlation analysis between the accumulated datasets and the distinct clinical standard deviations associated with each examining clinician. Such an analysis was crucial in elucidating potential disparities or uniformities in measurement techniques and outcomes across various clinical practitioners.

The empirical findings from this detailed examination revealed notable congruencies in the standard deviation metrics, both within the parameters of the proposed analytical model and the clinical data amassed from individual examiners. This congruence indicated a certain level of methodological consistency in the nuchal translucency measurements across diverse clinical settings (Table 1).

**Table 1:** Standard deviations in nuchal translucency (NT) measurements acquired from a 3D printed model and in a clinical setting (*p* = 0.906)

	Examiner 1	Examiner 2	Examiner 3	Examiner 4	Examiner 5	Examiner 6	Examiner 7	Examiner 8
Model NT standard deviation	0.660	0.460	0.080	0.240	0.380	0.360	0.540	0.180
Clinical NT standard deviation	0.700	0.040	0.360	1.020	1.100	0.120	0.080	0.260
Overall standard deviation	0.680	0.250	0.220	0.630	0.740	0.240	0.310	0.220

The table provides a comparative analysis of standard deviations in nuchal translucency (NT) measurements, contrasting data from a 3D printed model with clinical NT deviations extracted from GE ViewPoint version 6.0 (GE Healthcare, Solingen, Germany), thereby assessing measurement consistency across simulated and clinical environments

However, it is imperative to underscore that these observed similarities did not manifest statistical significance. The lack of statistical significance in this context underscores a critical aspect—the observed correlations, while visually apparent, do not substantiate a definitive or generalizable trend within the scope of statistical reliability. This observation, though seemingly a constraint in the study’s conclusions, serves as a catalyst for future empirical inquiries. It underscores the exigency for more extensive and methodologically robust follow-up studies. Such studies would be essential to probe deeper into the observed phenomena, potentially uncovering latent factors or variables that influence these outcomes. Therefore, while the study augments the existing corpus of prenatal diagnostic research, it concurrently establishes a foundation for future scholarly exploration in this domain.

### Cost effectiveness

The combined production of the abnormal and physiological models consumed 28 ml of resin in our 3D printer. The overall printing duration, conducted concurrently, amounted to 8 h and 15 min. The expenses associated with the High Temp Resin V2 (priced at 236.81 euros per liter) totaled 6.63 euros for both models. Isopropyl alcohol (99%, 10 L for 35.95 euros) was utilized for model rinsing, with potential for recycling. Inclusive of electricity costs for the printer, Formlabs Cure and Wash apparatus, and factoring in the expense of latex ultrasonic sleeves (condoms) at approximately 16 cents each (0.32 euros for two), along with glue priced at 8.89 euros, the direct material costs (excluding reusable items like rinsing fluid) summed up to 6.95 euros for both models. It is imperative to consider the overarching expenses related to material procurement, electricity consumption, and equipment maintenance when assessing the comprehensive production costs.

## Discussion

The contemporary landscape of medical education and diagnostic practices is continually evolving, with an increasing emphasis on the integration of innovative technologies to enhance training and proficiency in various medical disciplines. Our recent study addresses this paradigm shift by introducing a novel approach to ultrasound model construction, specifically focusing on the replication of sonographically detectable NT in 3D printed fetal models.

The relevance of realistic simulation tools in ultrasound education has been emphasized in recent scientific literature [23]. Tolsgaard et al. discussed the challenges in ultrasound education and underscored the need for innovative training tools that bridge the gap between theoretical knowledge and practical skills [9, 24]. Furthermore, Dietrich et al. highlighted the critical role of simulation in improving diagnostic proficiency in prenatal ultrasound [25]. Our study contributes to this discourse by demonstrating the utility of 3D printed fetal models in providing a hands-on experience for ultrasound novices to acquire and for experienced clinicians to refine their NT assessment skills, a critical parameter in prenatal screening. The potential impact of improving training and subsequent diagnostic accuracy for direct patient outcomes cannot be understated.

The accuracy and reliability of our models are evident in the correct classification by a diverse group of specialists. This consistency in measurements and classifications across different levels of expertise attests to the robustness of our approach and suggests that the technology could be successfully integrated into various medical training programs. However, a nuanced interpretation of the data is essential. Despite minor deviations in individual judgments from the respective model values, there is a remarkable consensus among the examiners in accurately discerning the inherent differences or gradients within the models. This precision in recognizing the differences contributes to the observed high values in the interrater reliability (ICC correlation). Evans et al. demonstrated that even minor inaccuracies in NT measurements, as minimal as 25% or 0.5 mm, can significantly compromise abnormality detection [26]. Such discrepancies lead to an 18% reduction in detection rates, decreasing from 81.7% to 67.1%. Just as the importance of standardization and quality assurance is universally acknowledged in laboratory measurements, the same level of rigor should be applied to NT measurements, given their integral role in algorithmic applications [5].

Our model is suitable as a reference system to show the individual deviations that each examiner has within the scope of the NT measurement. The 3D model thus serves as a sonographic measurement feedback tool for the individual examiner and shows them how accurate their measurements are.

However, Braithwaite et al. demonstrated that even machine–probe combinations can yield both statistically and clinically significant variations in measurements, particularly pertinent to NT measurements in clinical practice [27]. This principle extends beyond NT measurement to other ultrasound-based obstetric parameters. These variations may contribute to discrepancies in NT measurements during audits of sonographers or centers, reflecting both real differences in ultrasound equipment and its often inadequate calibration for NT measurement accuracy. Importantly, these findings emphasize the need for cautious interpretation of NT screening results, as our observations suggest that expectations for measurement precision may surpass realistic achievable accuracy levels.

It is noteworthy to consider the cost-effectiveness of our method. The ultrasound phantoms available on the market represent pregnancies in the 20th week of pregnancy for practicing biometry. Nuchal translucency cannot be measured on these models due to the advanced week of pregnancy. In addition, none was constructed in this model. Another disadvantage of this ultrasound model is the price of 11,125.31 euros [28]. Tolsgaard et al. emphasized the importance of cost-effective solutions in medical training and diagnostic practices [29]. Our study aligns with this perspective, showcasing how 3D printing technology provides an economical alternative for creating realistic fetal models and promoting accessibility to advanced training tools, which potentially democratizes medical education [30]. However, this accomplishment not only affirms the authenticity of our 3D printed fetal models in replicating intricate fetal anatomical features crucial for prenatal diagnosis, but also represents the superiority over two-dimensional images [31].

## Limitations

First, while our 3D printed fetal models accurately replicated the sonographically detectable NT and demonstrated high inter-rater reliability, the generalizability of the findings may be influenced by the specific characteristics of the healthy volunteer used for model creation. The anatomical variations inherent in different populations and pregnancies could impact the fidelity of the models in representing diverse clinical scenarios.

Second, the study focused on the training aspect and diagnostic utility of the 3D printed fetal models, emphasizing their role in enhancing clinicians' proficiency in NT assessment. However, the direct translation of improved skills into clinical practice, long-term training effects and patient outcomes remains an area for further investigation. The study primarily addressed the accuracy of NT measurement and the ability to distinguish between physiological and abnormal models, and future research could explore the impact on clinical decision-making and patient care.

Additionally, the cost-effectiveness analysis presented in the study considered direct material costs, such as resin, isopropyl alcohol, and latex ultrasonic sleeves. However, a comprehensive economic evaluation should include indirect costs related to equipment maintenance, electricity consumption, and training. Future studies could delve deeper into the long-term economic implications and return on investment associated with implementing 3D printed fetal models in medical training programs.

Another aspect is that the model does not meet all FMF requirements. Besides that, fetal movements and positions of hyperflexion and hyperextension that can make measuring NT difficult were not present. For example, no brain structures were constructed in the skull. In the further development of the model, an attempt was made to add a cerebrum for better sagittal adjustment. Various resins were tried out here. This was not pursued further due to sound cancellation caused by additional structures. For follow-up projects, other materials should possibly be tried out to optimize the models. Furthermore, the study utilized a single 3D printer and a specific type of resin. While this ensured consistency in the printing process, the generalizability of the findings to other 3D printers and materials needs consideration. Variations in printing technologies and materials may influence the reproducibility of the models in different settings.

## Conclusion

By introducing a practical, cost-effective and accurate method for creating ultrasound models, we contribute to the ongoing conversation about the integration of cutting-edge technologies in medical training. The positive outcomes and alignment with recent literature underscore the potential of 3D printed fetal models to contribute to medical education and enhance diagnostic proficiency in the field of prenatal ultrasound. Furthermore, the 3D model not only serves as a training tool for prenatal ultrasound novices but may also function as a feedback tool for experienced examiners, providing insights into the (long-term) accuracy of their sonographic NT measurements. Future studies could explore the broader applications of this technology and its impact on clinical outcomes, paving the way for a new era in medical training and practice.

## Abbreviations

3D: Three-dimensional
FMF: Fetal Medicine Foundation
NT: Nuchal translucency
CRL: Crown-rump length
SD: Standard deviation
ICC: Intra-class coefficient
ANOVA: Analysis of variance
